# Agricultural Crops Grown in Laboratory Conditions on Chernevaya Taiga Soil Demonstrate Unique Composition of the Rhizosphere Microbiota

**DOI:** 10.3390/microorganisms10112171

**Published:** 2022-10-31

**Authors:** Irina Kravchenko, Mikhail Rayko, Ekaterina Tikhonova, Aleksey Konopkin, Evgeny Abakumov, Alla Lapidus

**Affiliations:** 1 Winogradsky Institute of Microbiology, Research Center of Biotechnology, Russian Academy of Sciences, 119071 Moscow, Russia; 2 Center for Bioinformatics and Algorithmic Biotechnology, St. Petersburg State University, 199034 St. Petersburg, Russia; 3 Department of Applied Ecology, Faculty of Biology, St. Petersburg State University, 16th Liniya V.O., 29, 199178 St. Petersburg, Russia

**Keywords:** metagenomics, rhizosphere microbiome, Chernevaya taiga, Umbrisol, Retisol, plant gigantism, plant growth promoting rhizobacteria (PGPR)

## Abstract

Chernevaya taiga in West Siberia is a unique environment, with gigantism of grasses and shrubs. Exceptionally high productivity of plants is determined by the synergistic interaction of various factors, with a special role belonging to microorganisms colonizing the plant roots. This research explored whether agricultural plants can recruit specific microorganisms from within virgin Chernevaya Umbrisol and thus increase their productivity. Radish and wheat plants were grown on the Umbrisol (T1) and control Retisol of Scotch pine forest stand (T3) soils in the phytotron, and then a bacterial community analysis of the rhizosphere was performed using high-throughput sequencing of the 16S rRNA genes. In laboratory experiments, the plant physiological parameters were significantly higher when growing on the Umbrisol as compared to the Retisol. Bacterial diversity in T1 soil was considerably higher than in the control sample, and the principal coordinate analysis demonstrated apparent differences in the bacterial communities associated with the plants. Agricultural plants growing in the T1 soil form specific prokaryotic communities, with dominant genera *Chthoniobacter, Pseudomonas, Burkholderia,* and *Massilia.* These communities also include less abundant but essential for plant growth nitrifiers *Cand. Nitrosocosmius* and *Nitrospira,* and representatives of Proteobacteria, Bacilli, and Actinobacteria, known to be gibberellin-producers.

## 1. Introduction

According to forecasts, the most important problems for humanity in the current century will be global warming, the decline in biodiversity and ecosystem sustainability, and the lack of energy and food. Long-term environmental conditions would have severe impacts on agricultural production, food security, availability, and accessibility [1]. Rapidly expanding human population and economic growth increased pressure on biodiversity resources, and up to one-half of the terrestrial surfaces have been altered by human activity [2]. Only 2–3% of the Earth’s terrestrial surface remains ecologically intact [3], and Chernevaya taiga (tall-grass fir-aspen forest) is an example of an undamaged wilderness area. 

Chernevaya taiga is a unique forest ecosystem, unaffected by anthropogenic impact, located in the Altai-Sayan mountain region of Siberia, Russia. It is a small area with naturally isolated micro-habitat, a boreal formation at elevations ranging from 400 to 900 m above sea level [4]. Due to the amount of snow in the winter and the overall amount of precipitation throughout the year, the soil of Chernevaya taiga never freezes, resulting in a level of biodiversity comparable to those found in tropical rain forests [5]. These forests are characterized by the presence of relict flora species and gigantism of herbaceous plants. These features are usually associated with the local hydrothermal and climatic conditions, related to the rapid cycling of nutrients within the ecosystem [6]. 

One of the characteristic distinguishing features of Chernevaya taiga that separates it from other Siberian forests is that the stand is dominated by Siberian fir (*Abies sibirica*) and aspen (*Populus tremula*) without a clear predominance of one over the other [7]. The most notable unique ecological characteristic of Chernevaya taiga is gigantism of the perennial grass plants and shrubs. Large shrubs up to 4.5 meters, of *Sorbus sibirica*, *Padus avium,* and *Salix caprea*, do not form a closed canopy. The well-developed closed grass cover of the taiga is formed by such giant (up to 2.5 m) perennial plants as *Saussurea latifolia*, *Crepis sibirica*, *Aconitum excelsum*, *Cacalia hastata*, *Pleurospermum uralense*, *Bupleurum aureum*, *Lathyrus gmelinii*, *Aconitum septentrionale*, *Delphinium elatum*, and *Thalictrum minus.* This area is also characterized by mass-flowering early spring plants and ephemerides, the presence of species classified as Pliocene relics, and the almost complete absence of moss cover [8]. Despite the flushing type of water regime, leaching the organic matter from the soil profile, soil fertility remains very high, significantly exceeding the fertility of agricultural lands [6]. 

The rhizosphere, the interface between plant roots and surrounding soil, is an active component of plant–soil systems [9]. It contains a specific microbial community that can influence plant growth, and can be viewed as a “root microbiome” [10,11]. Rhizosphere microorganisms actively participate in plant nutrient absorption, protecting against pests and pathogens and increasing resistance to stress [12]. Plants attract soil microorganisms through root exudates and root litter, forming a rhizosphere community with microorganisms of various trophic levels [13]. In addition to substrates for the growth of microorganisms, root exudates also contain signaling molecules, microbial attracts, stimulants, inhibitors, or repellents [14].

Little is known about the factors determining the composition of the rhizosphere microbial communities. The changes in the rhizosphere community’s composition were found during the development of the host plant [15]. Differentiations of bacterial communities at the rhizosphere–soil–root interface were revealed [16,17,18]; however, to date the underlying mechanisms of differentiation are unknown. In addition, although the effect of root exudates on rhizosphere communities is well known [19], there is no information about the dynamics of such changes.

It was shown that the functionality of the rhizosphere microbiota depends on the presence of certain species, but recent studies demonstrated that it is determined by the interaction between organisms more than by the presence of a certain species [20]. This transition to functional analysis of the rhizobiome can significantly expand the understanding and manage of the rhizosphere microbiome. Functional diversity at the microbial community’s level is well documented and can be associated with the ecology of the community [21].

Plant growth and development is stimulated by environmental signaling molecules or internal molecules such as hormones. Many of the plant growth promoting rhizobacteria (PGPR) are capable of producing hormones and thus may play a significant role in plant growth. For example, PGPRs synthesize indole-3-acetic acid (IAA), a phytohormone that affects various processes in plants [22]. At a low concentration of exogenous IAA, elongation of primary roots occurs, while high levels of IAA, on the contrary, reduce the length of the primary root, but stimulate the formation of lateral roots and root hairs [23]. Another group of phytohormones, gibberellins (GAs), stimulates the division of plant cells, controls the differentiation of the root meristem, and induces the proliferation of root hairs [24].

Some rhizobacteria, such as *Rhizobium* and *Azospirillum*, are known to produce GA [25]. Production of gibberellin-like substances has also been found in other bacterial genera, including *Acetobacter*, *Herbaspirillum,* and *Bacillus* [26].

We hypothesized that the plant root system might recruit soil microorganisms carrying specific traits of growing plants. The present study aims to assess differences/similarities between the rhizosphere microbiota of agricultural plants growing on Chernevaya taiga Umbrisol and control Retisol in the laboratory pot experiments. Here, we characterize bacterial communities by 16S rRNA high-throughput sequencing. In addition, we measured the cultivable bacteria number, plant physiological parameters, and physicochemical soil parameters.

## 2. Materials and Methods

### 2.1. Regional Settings, Sampling and Laboratory Analysis

The field study was conducted in May 2020 on the territory of the Tomsk region, Russia. Soils from two environments were selected for this study, namely T1, Chernevaya soil-dark gray soil (Umbrisol, Albic, Loamic, Folic WRB classification [27]) under the tallgrass fir-aspen forest with a shrinking fir stand (56.30693 N, 85.47063 E) and T3, control soil–oligotrophic sandy loam soil (Retisol, Luvic, Folic WRB Classification) under the pine forest with an admixture of larch (56.48106 N, 84.79860 E). A detailed description of the areas and study sites were given in the previous publications [28,29]. 

Various soils compose the soil cover of the Chernevaya taiga ecosystem [28]. The most typical soils for this environment are Umbrisols and Retisols. Chernevaya taiga is confined to landforms with heights ranging from 200 to 700/800 m asl. Normally, these ecosystems are located on the western, windward slopes of the mountains and foothills of the southern part of Western Siberia. A unique combination of biotic, lithogenic, relief, and climatic soil formation factors leads to the formation of peculiar soils of Chernevaya taiga. These soils do not freeze in the winter and have essential storage of water throughout the year, which provides the rapid mineralization of the organic matter and the fixation of mineral nutrients in the topsoil of the soil section. The accumulation of biophilic elements and very intensive biogeochemical turnover of alkaline cations are the most important properties of Chernevaya taiga soils [30], which are associated with the phenomenon of gigantism and extremely high plant productivity. 

The profile of the Umbrisol consisted of the following horizons: O-AU-AUe-BI-BC-C sub-layered silty loams of Holocene slops. The Luvic Retisol profile is essentially shorter and consists of fewer horizons—O-AY-AC-C, where the C horizon is presented by sandy textured parent materials of aeolian late Holocene origin. 

General physicochemical characteristics are described in Table 1. Bulk soil samples for the physicochemical analyses were selected from 10 cm layer in quadruplicate; they were ground in laboratory and passed through 2 mm sieve. For microbiological research, each field sample was placed into sterile bag and stored on dry ice before further processing. The laboratory samples were processed immediately after arriving to laboratory.

Soil pH values were determined potentiometrically in suspension with water to soil ratio of 2.5:1 (Seven CompactTM, Mettler, Germany). The particle-size composition of soils was determined by the commonly adopted Kaszynski sedimentation method. Total carbon (C) and nitrogen (N) content in soil samples were estimated with an elemental analyzer (LECO CHNS-932, Manasquan, NJ, USA) by dry combustion. Nitrate-NO_3_^-^ and ammonium-N NH_4_^+^ were extracted with 0.5 M K_2_SO_4_ and then analyzed by the indophenol method [31]. NH_4_^+^ was quantified directly in the soil extract, and NO_3_^-^ after reduction to ammonium by zinc powder and 10% CuSO_4_ solution. All analytical procedures were done in five replicates. Concentrations of total phosphorus and metals were measured by the atomic absorption spectrometer “Kvant-2MT” (Kortek, Russia).

### 2.2. Conducting Pot Experiments with Radish and Spring Wheat Plants

The experiment was conducted in February–March, 2021 in a phytotron at 23 ± 1 °C, 50–60% RH, 550 M photon/m^2^/s, and 16 h photoperiod. The soil in the plastic pots with non-sterile soils was kept at 60% water holding capacity by daily watering.

Surface-sterilized seeds of radish (*Raphanus raphanistrum subsp. Sativus*, cultivar “Red light”) and spring wheat (*Triticum aestivum L*., cultivar “Lada’’) were germinated on moistened filter paper for 2 days. Then ten seedlings were transferred to a pot containing 0.2 kg either Chernevaya or control soil. All experiments were done in three replicates. Data on plant height (mm), root length (mm), and the dry weight of shoots and roots (70 °C, 72 h, mg) were measured at the end of the growth period; the root to shoot ratio was also calculated.

For rhizosphere microbiota analysis, plants were destructively sampled at the end of the experiment, the whole root system was harvested and gently shaken to remove excess soil adhered to the root system. Then soil closely attached to the root system was collected by a vigorous vortex for 5 min in sterile PBS solution and centrifuged at 4000 rpm for 5 min. The supernatant was discarded, and one part of the soil fraction was stored at 4 °C for bacteria counting, while the other part was stored at −80 °C until subsequent DNA extraction. 

### 2.3. Bacterial Quantification

Two culture media, namely soil agar and Luria–Bertani (LB) agar, were used to quantify bacterial colony forming units (CFU) in rhizosphere soil. Rhizosphere soil fraction received as described in Section 2.2 was vortexed and serially diluted. A hundred microliters of the 10^−4^–10^−7^ dilutions were plated and incubated at 28 °C for seven days. Five technical replicates of the dilution series were plated for quantification. 

### 2.4. DNA Isolation, Amplification, and Illumina Sequencing of the 16S rRNA Gene 

Total soil DNA was extracted from 0.25 g of rhizosphere soil samples using the Power Soil DNA Isolation Kit (Qiagen, Calsbad, CA, USA). The NanoDrop 1000 spectrophotometer (Thermo Scientific, Waltham, MA, USA) was used to determine DNA concentration and purity.

Variable V4 region of the 16S rRNA gene was selected for analysis and amplified using universal primers 515F (5′-GTGCCAGCMGCCGCGGTAA-3′) and 806R (5′-GGACTACVSGGGTATCTAAT-3′) [32]. Library preparation and following high throughput sequencing was performed as described in previous study [28].

### 2.5. Bioinformatics Analysis

Preprocessing of the Illumina sequencing reads included removal of the adapters and indices using the Cutadapt program [33], as well as denoising, combining paired reads, and deleting chimeras using the Dada2 package [34] implemented in the QIIME2 package, v-2019.7 [35]. 

After filtering, high-quality amplicon sequence variants (ASVs) were obtained and included for subsequent analyses. The taxonomic classification of the obtained amplicon sequence variants was performed using Naive Bayes classifier trained on 99% OTUs sequences from Silva 138 database for the SSU rRNA genes [36]. All features annotated as mitochondria or chloroplasts were also removed. After filtering, on average 64,541 high-quality sequences per sample (min = 58,057 and max = 71,418) were obtained and included for subsequent analyses.

The ASV abundance of each sample was standardized by using the lowest level of sequence depth as a reference. To keep focus on “root generalists” and remove the possible PCR artifacts or chimeras, only features with an abundance of more than 10 reads and presented in rhizospheres of both plants in study (in any type of soil) were selected for subsequent analysis.

Further processing, including the construction of a phylogenetic tree using the FastTree algorithm [37], the calculation of α- and β-diversity, was performed within the QIIME2 package [35], and the plugins implemented in it, including standard diversity (Faith’s PD), evenness (Pielou’s index), and distance (UniFrac, Bray–Curtis dissimilarity) metrics.

The packages MicrobiomeAnalyst [38] and DESeq2LEfSe [39] were used to identify differentially abundant taxa and to visualize results. 

### 2.6. Nucleotide Sequence Accession Numbers

The raw data generated from 16S rRNA gene sequencing of microbial communities from rhizosphere soil samples have been deposited in the MG-RAST database as the “Chernevaya Taiga of Western Siberia-rhizosphere microbiome” project (mgp103638). 

## 3. Results

### 3.1. Soil Properties and CFU Data

pH values measured in soil-water suspension ranged from 6.3 in T1 soil to 6.2 in T3 soil (Table 1). Soils of Chernevaya taiga (T1) showed essentially higher content of total carbon (C) as compared with control forest soil: 9.9 and 2.4%, correspondingly. Similar results were observed for the contents of the soil total nitrogen (N). 

Parameters of texture, the content of sand, silt, and clay are very different for T1 and T3 soils. First, there is an essential difference in clay content, which is higher in T3 soil. The silt content in T1 is more than two times higher, as compared to T3 (Table 1).

Soil contents of total carbon, nitrogen, phosphorus, and potassium and C/N ratio were higher in T1 than in T3 (Table 1). Furthermore, higher levels of Fe, Mg, and Zn were found in T1.

We have found the bacterial CFU number ranging from 8.1 ± 1.1 × 10^7^ to 9.5 ± 2 × 10^8^ in 1 g of wet rhizosphere soil. The bacterial abundances in the rhizosphere were significantly higher for radish plants (9.5 ± 2 ×10^8^ and 6.8 ± 1.1 ×10^8^ CFU) compared with wheat (8.1 ± 1.1×10^7^ and 1.0 ± 0.6 ×10^8^ CFU) (*p*-value ≤ 0.05) when growing on the Chernevaya T1 and control T3 soils, respectively.

### 3.2. Plant Physiological Parameters and Rhizosphere Bacterial CFU Number for Artificially Grown Plants

**Figure 1:**
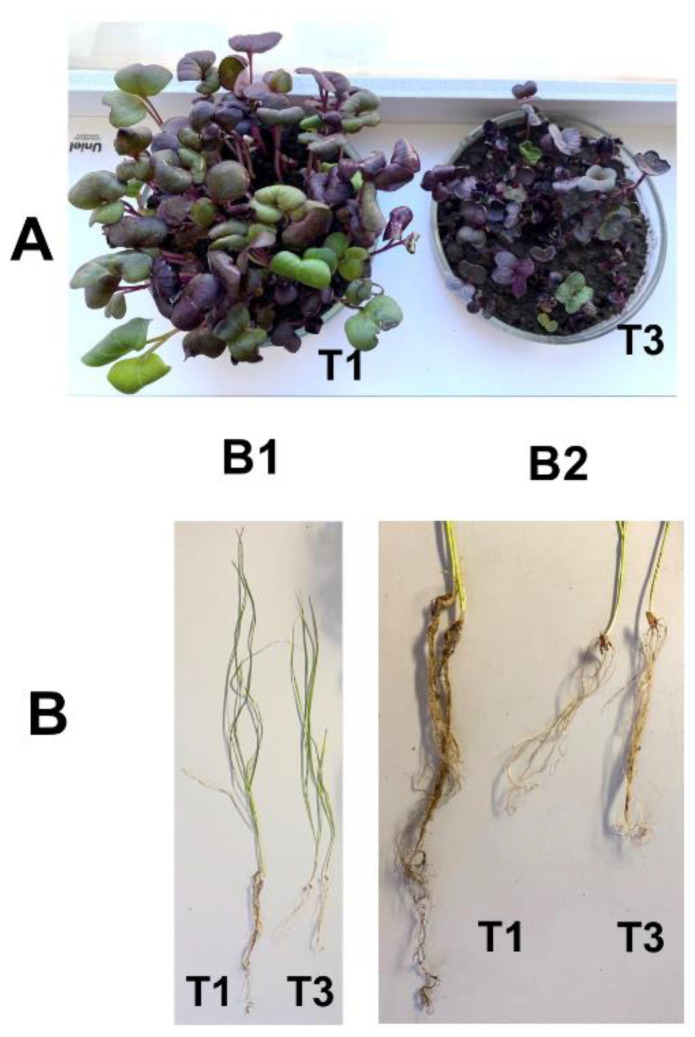
Phenotype and plant traits of radish (**A**) and spring wheat (**B**). B1: general view of wheat plants used in the experiment; B2: details of their root system. Plants were grown in the pot experiment with Chernevaya soil (T1) and the control soil (T3) samples.

Physiological parameters of the plants grown on the Chernevaya soil (shoot height, root length, and dry mass) were significantly higher compared to those grown on the control soil (Figure 1, Table 2). 

In the pot experiment, soil type had a significant effect on plant morphological characteristics, plant height, and root length (Figure 1). At the same time, total root length was three to five times longer in wheat plants and twice as much in radish plants. The total plant dry mass was significantly (*p* < 0.05) greater in the T1 soil than in T3 soil for both types of plants (Table 2). 

### 3.3. Overview of Microbial Community Diversity

**Figure 2:**
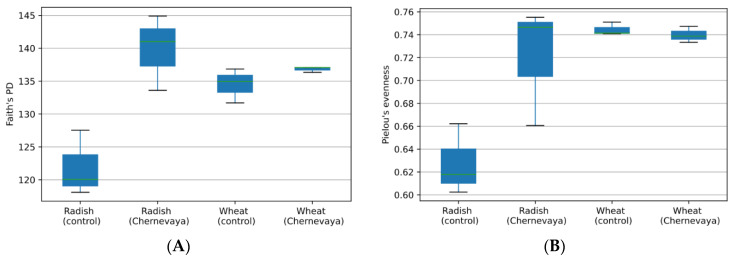
Diversity of the microbial communities in the rhizosphere of radish and wheat plants growing at the Chernevaya and control soils based on Faith’s phylogenetic diversity index (**A**) and Pielou evenness index (**B**).

To explore the species richness of a particular sample, we have calculated phylogenetic diversity using the Faith’s PD index (Figure 2A), and species evenness using Pielou’s index (Figure 2B). We observed increased diversity in Chernevaya taiga soils compared to control (ANOVA F-value 109.51, *p*-value: 7.7837 × 10^−7^). With the pairwise comparison, we observed increased species diversity in the rhizosphere communities of the radish plants in Chernevaya taiga soil samples (*p*-value 0.049), and no significant increase in wheat plants (*p*-value 0.12).

Analysis of evenness, or abundance of species relative to other species in a given community, showed no significant difference between samples. Species evenness index varied from 0.6 to 0.75 (Figure 2B). The rhizosphere communities of the radish plants on control soils demonstrated the most uneven species composition, but the pairwise comparison *p*-value was below the significance threshold 0.05.

**Figure 3:**
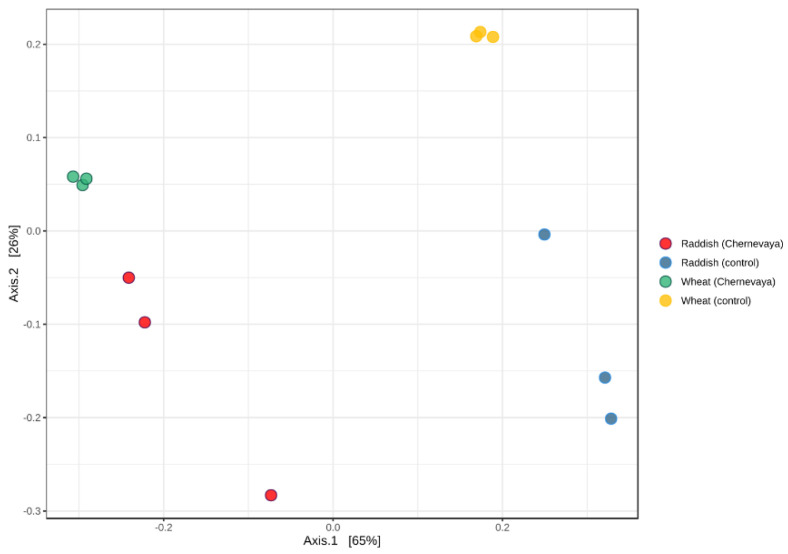
Analysis of beta diversity structure of rhizosphere microbiome samples visualized by principal component analysis (PCA) using Bray–Curtis dissimilarity index.

Beta diversity metrics were analyzed to elucidate major drivers of microbial community composition using the weighted UniFrac distance metric, based on species content and phylogenetic information. Principle coordinate analysis (PCoA) consistently indicated soil type as the vector responsible for the greatest amount of variability. Our results show that microbial communities in the rhizosphere in both plants on Chernevaya taiga soil demonstrated pronounced clustering clearly separated from those in control soil (Figure 3). Interestingly, communities of radish and wheat grown in Chernevay soil were clustered together. On the control soil, the communities formed separate clusters (Figure 3).

### 3.4. Microbial Community’s Composition in Rhizosphere of Radish and Wheat Plants 

**Figure 4:**
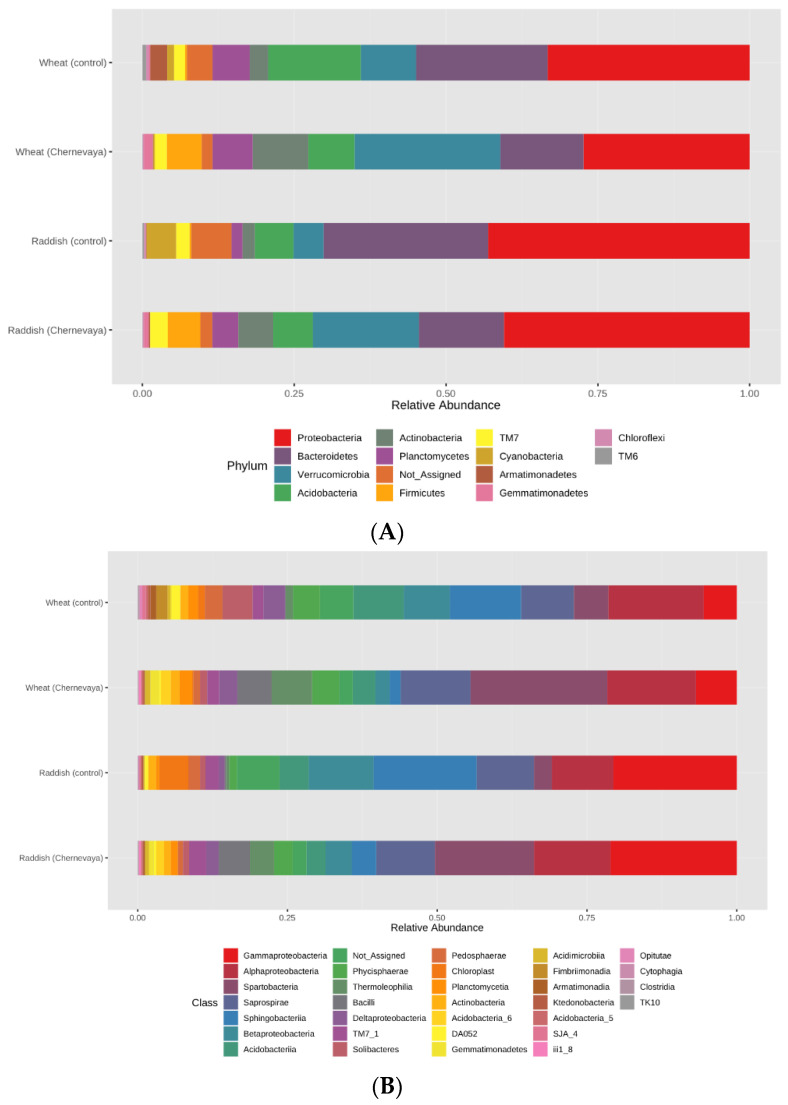
Relative average abundances of the prokaryotic taxa in the rhizosphere of radish and wheat, growing on the T1 and T3 soils in the laboratory experiment on the phyla (**A**) and classes (**B**) level. The values were summarized at phylum level (keeping only phyla with relative abundance >1%) and relativized to 100%. See Supplementary Table S1 for the abundance values for each taxonomic level.

The revealed differences in α- and β-diversity of rhizosphere prokaryote communities prompted us to study more deeply the differences in the taxonomic composition and relative abundance of bacterial taxa. All studied samples had a similar structure at the phylum level and contained representatives of 18 phyla typical for soils. Proteobacteria, Bacteroidetes, Acidobacteria, Verrucomicrobia, and Actinobacteria were the five dominants in all rhizosphere soil samples (Figure 4A), and the proportion of unclassifiable microorganisms was also very high. At the same time, the relative abundance of the most common phyla differed significantly for plants grown in T1 soil and T3 soil (Figure 4A). At the phylum level, Actinobacteria and Verrucomicrobia were most abundant, and Archaea and Firmicutes were present exclusively in the rhizosphere of plants growing in Chernevaya soil. Planctomyces were found only in the rhizosphere of wheat plants. At the class level, Spartobacteria (Verrucomicrobiota), Bacilli (Firmicutes), and Chloracidoacteria (Acidobacteria) dominated in the rhizosphere of the T1 soil growing plants, while Sphingobacteria (Bacteroideta), Acidobacteria, and Actinobacteria prevailed in the control soil (Figure 4B, Supplementary Table S1). The most common genera in the rhizosphere of T1 soil were *Chthoniobacter, Pseudomonas, Bukholderia,* and *Massilia*. Thus, the soil has a significant impact on the diversity and structure of the microbial community of the rhizosphere.

### 3.5. Evaluation the Differentially Abundant Prokaryotic Taxa

Between-classes PCA of the whole community structure showed that soils had specific effects on the rhizospheres’ microbiomes of the radish and wheat plants. The dissimilarity in the rhizosphere community structure between the Chernevaya and control soils can largely be attributed to those most strongly selected or depleted taxa. 

**Figure 5:**
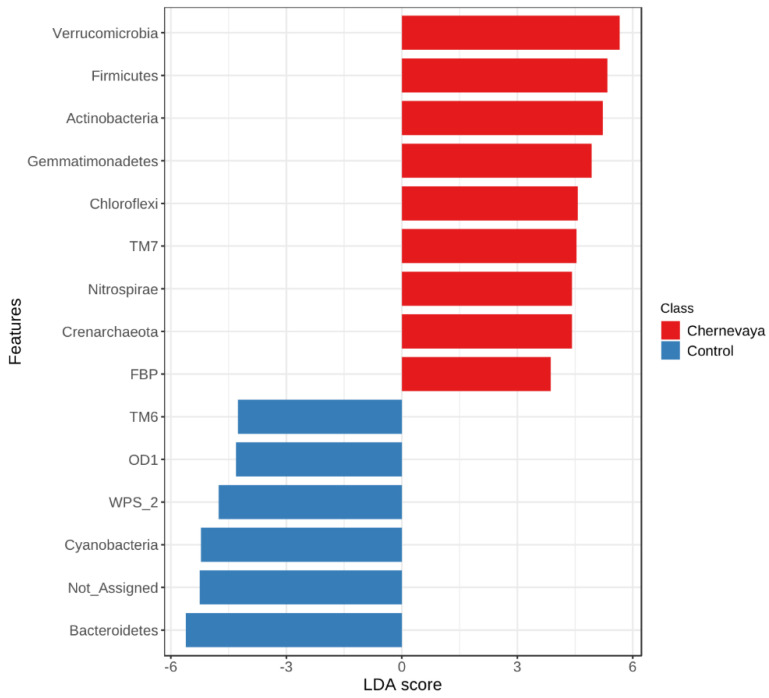
Effect size (LDA) for the prokaryotic phyla in the rhizosphere of plants growing on the Chernevaya soil relative to those on the control soil. Only taxa recruited in rhizospheres of all plants in study (“root generalists”) were taken into account. LDA score is a log10 transformed, absolute threshold 2, FDR-adjusted *p*-value cutoff 0.05. See Supplementary Table S2 for the analysis result for each taxonomic level.

To evaluate prokaryotic taxa which may be less abundant but be of great importance in the plant growth, we performed linear discriminant analysis and calculated the log10 LDA score for each taxon in each sample. We ranked differentially abundant bacteria to identify bacteria with the most substantial population responses to the growth in the Chernevaya soil (Figure 5, Supplementary Table S2). Responses in both positive (more abundant) and negative (less abundant) directions were observed.

In this work we focused on the taxa that can provide high plant growth in taiga soils for different plants. For this purpose, we selected those taxa that are present in the rhizosphere of both plants (the so-called “root generalists”). As a result, we obtained a dataset of 231 genera. The majority of the prokaryotic sequence variants with a high degree of selection in the rhizosphere of Chernevaya soil compared to the control are the same for wheat and radish plants. The most selected were the phyla Verrucomicrobiae, Firmicutes, Actinobacteria, Gemmatimonadetes, Chloroflexi, Saccharibacteria (TM7), Nitrospirae, and Crenarchaeota. Additionally, members of Candidate phylum Gracilibacteria GN02 were highly selected in the wheat rhizosphere and Verrucomicrobiae in the radish rhizosphere.

**Figure 6:**
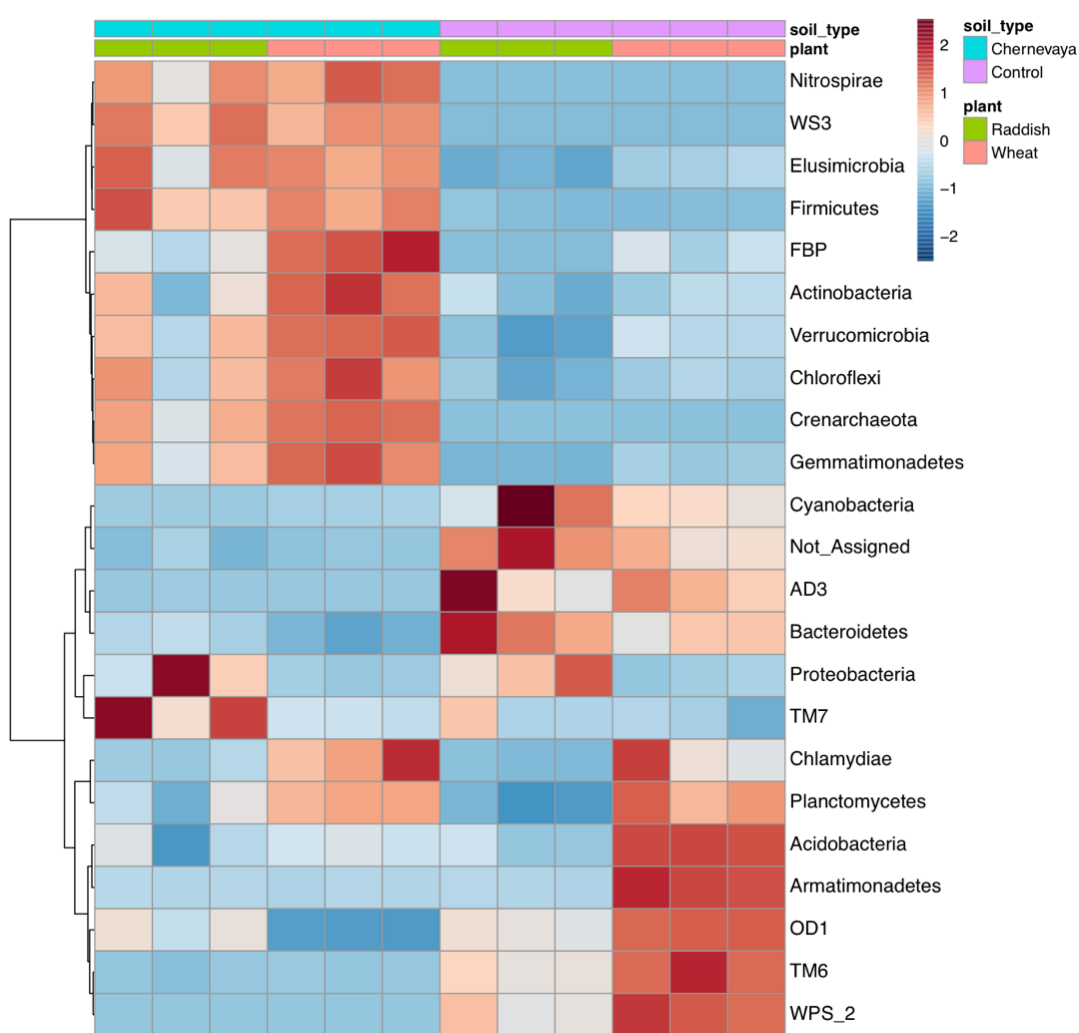
Heatmap diagrams showing the hierarchical clustering of the rhizosphere communities on the phylum level. Euclidean distance, Ward’s linkage clustering method.

Hierarchical clustering of the rhizosphere communities showed clear separation of the Chernevaya and control soil samples (Figure 6).

## 4. Discussion

The rhizosphere is a root zone considered as a hotspot for bacterial diversity, where bacteria are mainly expressed in functions adapted to the root’s presence and promote plant growth. During the growth process, plant roots secrete carbon compounds that are used by rhizosphere microorganisms. In turn, microorganisms affect plant growth by increasing the availability of mineral compounds and forming biologically active substances [13]. Plant-associated microbes can be recruited from the environment or from seeds. However, most of the beneficial microorganisms fall into the rhizosphere from the soil [40]. The current study aimed to evaluate whether the rhizosphere microorganisms may be the drivers of the extremely high productivity of plants growing on the Chernevaya soil. We intended to answer the questions of (1) whether agricultural plants growing in the laboratory experiments with Chernevaya taiga soil manifest the high growth parameters and (2) whether there are specific features of the rhizosphere microbial communities which would be likely to control the plant gigantism. 

In general, the answer to both questions is yes. The physiological characteristics of wheat and radish plants grown on T1 soil significantly exceed those on the control soil. One of the reasons may be the high carbon and nitrogen content in the soil as well as metal (copper, iron, magnesium, zinc) contents. Iron, copper, and zinc are essential for plant cell metabolism, due to their ability to donate and accept electrons and be the components of proteins catalyzing redox reactions. Additionally, zinc is the cofactor of RNA polymerase [41], while magnesium participates in photosynthesis and associated physiological processes required for optimal plant growth and yield [42]. 

Alpha and beta diversity metrics suggest that rhizosphere samples of T1 soil hosted the more complex microbial communities, but only a few microbial phyla dominate. Rhizosphere microbial communities of wheat and radish growing in T1 soil were separated clearly from those in control soil. These data confirm the finding that bacterial rhizosphere phyla are conserved mainly among plants [43], and the variance in rhizosphere microbial communities is typically larger in the same plants growing in the different soils as in the different plants growing in the same soil [44]. The unique Chernevaya Umbrisol may be considered as a significant reservoir for microbial diversity, and the plant does appear to drive the differentiation of a small subset of soil microbiota. The presence of distinct microbial taxa in T1 and T3 rhizospheres propose that soil conditions (e.g., pH, texture, content of organic matter, metals) likely play an important role in microbial community structure, and define how the bacteria will be affected by the rhizosphere conditions.

Proteobacteria is the most abundant bacterial phylum in the rhizospheres under study, which is consistent with previous investigations of other soil systems [45]. Proteobacteria contains numerous PGPR genera, including *Pseudomonas*, *Azotobacter*, *Azospirillum*, *Rhizobium*, and *Enterobacter,* with promising applications for crop improvement. Other dominant rhizosphere phyla (˃5% of the sequences) were different across plants growing in Chernevaya and control soils. Verrucomicrobia and Actinobacteria dominated in T1 soil and contain different PGPRs with a variety of functions benefiting plant growth, while in T3 soil Acidobacteria and Bacteroideta prevailed. The growing interest to Actinobacteria application in agricultural practice is due to their ability to colonize root system of plants and, forming spores, to survive for a long time in unfavorable conditions [46]. Recent studies demonstrated that Verrucomicrobia abundant in rhizosphere form close associations with plant roots and have characteristics typical for PGPB [47]. 

According to modern conceptions, non-dominant taxa with a minor contribution to the whole community are recognized for their importance in maintaining alpha diversity in microbial communities and key ecosystem functions [48]. Possible roles of the rare taxa may include nutrient cycling, resistance to disturbance or invasion, and driving the functions of host-associated microbiomes [49]. Rare microbes may perform key functions that are costly for dominant taxa. For example, they play an essential role in soil nitrogen fixation [50], providing evidence for the potential disconnect between abundance and functional capacity. Bacteria with the substantial population responses to the growth in the Chernevaya soil were mostly the same for wheat and radish rhizospheres. The most selected were Chloracidobacteria (Acidobacteria), proposed class Ellin 6529 (Chloroflexi), Thaumarchaeota (Crenarchaeota), Nitrospira (Nitrospirae), Gemm-1 (Gemmatimonadetes), MB-A2-108 (Actinobacteria), and TM7-3 (Bacteroidetes). At the same time, only in the wheat rhizosphere GKS2-174 (Candidate phylum Gracilibacteria) was highly selected. 

It is necessary to note that the taxa with the highest degree of selection belong to difficult-to-cultivate organisms, many of which are absent in laboratory culture and their characteristics are unknown. Ellin 6529 is frequently distributed, but an understudied representative of the Chloroflexi [51]. Gracilibacteria is a candidate phylum included in the Patescibacteria group. Based on the genomic analyses it was suggested that Gracilibacteria has limited metabolism and may be symbionts [52]. Gemmatimonadetes contained only one valid species, but numerous environmental 16S rRNA gene sequences were found in the soil and marine environments [53].

One of our exciting findings was that nitrifying archaea and bacteria were found in the rhizosphere of plants growing in T1 soil. Ammonia-oxidizing bacteria and archaea are particularly relevant because of their roles in nutrient cycling in ecosystems. Archaea in the rhizosphere of the Chernevaya soil were classified as *Candidatus Nitrososphaera*. They mediate the first and rate-limiting step of nitrification, the conversion of ammonium to nitrate. We have also detected nitrite-oxidizing bacterium *Nitrospira*, a genus in Nitrospiraceae. Certain *Nitrospira* species are known as “completely” nitrifying (comammox) that perform both steps of ammonia oxidation [54]. *Nitrospira* were reported to be widely presented in terrestrial ecosystems and were found in bulk Chernevaya soil [28], and were recently discovered in the rhizosphere of perennial grasses [55].

Currently, the agricultural sector of the economy is oriented towards environmentally sustainable development, which means increasing productivity while preserving natural resources and the environment [56]. In this regard, there is an ever-growing interest in the study of soil and rhizosphere microorganisms that can be used to increase crop yields [57]. Long-term land usage in agricultural practice leads to negative implications such as disturbances of the global nutrient cycles, degradation of organic matter, increased greenhouse gas emissions, reduced soil fertility, etc. [58]. Agricultural soils are characterized by low microbial taxonomic and functional diversity, and domestication may have affected plant phenotypes relating to the recruitment of beneficial microorganisms from the soil [59]. From this point of view, Chernevaya taiga is a unique ecosystem that preserves the “pre-agricultural” microbiome, not affected by agricultural practices. Most investigations focus their attention on the effects of PGPR use on the yield of agricultural crops [60]. The idea of using PGPR from a unique environment, known for its high productivity is, however, novel.

Despite its unique properties, information about the microbial communities of Chernevaya soil is very limited. Based on previously conducted metagenomic studies the taxonomic composition of microbial communities in Chernevaya taiga soil differs significantly from those of the soils of the surrounding forest areas [28]. On the other hand, this object may be of great importance in the search of PGPR, cellulolytic microorganisms, as well as producers of antibiotics, auxins, and other biologically active molecules. In addition, understanding the causes of gigantism can be used to increase crop yields by environmentally friendly techniques. The impact of soil microbiome on ecosystem biodiversity, productivity, and stability has been recognized [61,62,63], although studies explaining and analyzing such effects are few, compared with the significance of this global problem. Knowledge of the distribution and diversity of indigenous bacteria in the natural ecosystem with high fertility, their isolation and characterization can be the basis for creating agricultural biotechnology based on the novel PGPR strains. 

## 5. Conclusions

Identification, characterization, and further isolation of PGPR from the soil of the unique environment, Chernevaya taiga, may represent excellent novel biotechnological tools. Considering that soils for vegetable production are limited, the use of these bacteria would improve the tolerance, productivity, and yield of plants of agronomic interest which is a problem for world food security. 

In conclusion, our experimental data contribute to an understanding of how soil microorganisms and plants create specific root-associated microbial communities. This study is the first attempt to elucidate the bacterial diversity and composition in the rhizosphere of the crops artificially growing on the Umbrisol using high-throughput sequencing methods. We have demonstrated that the rhizosphere microbiota of agricultural plants grown on Chernevaya taiga soil differs from corresponding microbiota of the control soil, and that differences were revealed at the level of dominant taxa and minor components of the community. It was found that significant enrichment of rare, mainly uncultured prokaryotic taxa occurs in the rhizosphere. The fact that similar microorganisms from Chernevaya taiga soil are highly enriched in different crops may suggest their significance for plant growth. For some components of rhizosphere consortia, the role of nitrifying archaea and bacteria in stimulating plant growth is clear, whereas, for others, it requires further research.

Our promising result supports the idea that the bacteria, specifically for Chernevaya taiga soil, influences plant growth through the creation of the specific rhizobiome. A more detailed analysis of individual strains’ roles and combinations will shed light on this effect. Future studies should investigate the functional role of the root-associated microbial communities, including both isolates and uncultured microorganisms.

## Figures and Tables

**Table 1:** General soil parameters of experimental plots, average of 5 replicates +/− SE. The most significant differences (*t*-test, *p*-value ≤ 0.05) are highlighted in bold.

Unit	Item	Chernevaya Umbrisol (T1)	Control Retisol (T3)
%	Clay	39 ± 7	58 ± 16
	Silt	**42 ± 3**	19 ± 3.1
	Sand	19 ± 2	23 ± 3.0
	C	**3.46 ± 0.4**	0.36 ± 0.06
	N	**0.28 ±** **0.05**	0.04 ± 0.01
	pH_H2O_	5.96 ± 1.4	5.12 ± 0.7
	C/N	12.31 ± 3.5	8.62 ± 1.1
mg kg^−1^	P	**234 ± 26**	44 ± 9
	N-NH_4_^+^	**7.32 ± 2.2**	0.09 ± 0.01
	N-NO_3_^-^	8.59 ± 1.3	12.21 ± 2.4
	K	**91.7 ± 14**	20.3 ± 3.0
	Na	**6.3 ± 2.2**	2.8 ± 0.4
	Cu	**2.8 ± 0.8**	0.28 ± 0.12
	Fe	**169.4 ± 32**	80.5 ± 6.5
	Mg	**32.2 ± 7.5**	14.0 ± 1.6
	Mn	16.4 ± 2.3	16.1 ± 1.8
	Zn	**7.0 ± 1.6**	1.4 ± 0.3

**Table 2:** Measurements of the physiological parameters of radish and wheat plants grown in pots with control and Chernevaya taiga soils.

Culture 1	Parameters	Chernevaya Soil (T1)	Control Soil (T3)
Radish	Shoot height, mm	80 ± 11	50 ± 5
	Root length, mm	70 ± 8	35 ± 4
	Root: shoot ratio	0.88	0.70
	Dry mass, mg plant^−1^	16.63 ± 0.4	15.47 ± 0.3
Wheat	Shoot height, mm	19.7 ± 3.3	16.3 ± 2
	Root length, mm	46 ± 1.3	37 ± 2.0
	Root: shoot ratio	0.41	0.43
	Dry mass, mg plant^−1^	140 ± 0.05	90 ± 0.03

## Data Availability

The data are available upon request.

## Supplementary Materials

The following supporting information can be downloaded at: https://www.mdpi.com/article/10.3390/microorganisms10112171/s1, Table S1: Relative average abundance of the prokaryotic phyla in different plant rhizosphere and soil types, for each taxonomy level; Table S2: Effect size analysis (LDA) for the prokaryotic taxa in the rhizosphere of plants growing on the Chernevaya soil relative to those on the control soil.

## References

[1] Muluneh M.G. (2021). Impact of climate change on biodiversity and food security: A global perspective—A review article. Agric. Food Secur..

[2] Vitousek P.M., Mooney H.A., Lubchenco J., Melillo J.M. (1997). Human Domination of Earth’s Ecosystems. Science.

[3] Plumptre A.J., Baisero D., Belote R.T., Vázquez-Domínguez E., Faurby S., Jedrzejewski W., Kiara H., Kühl H., Benítez-López A., Luna-Aranguré C. (2021). Where might we find ecologically intact communities?. Front. For. Glob. Chang..

[4] Nazimova D.I., Danilina D.M., Stepanov N.V. (2014). Biodiversity of rain-barrier forest ecosystems of the Sayan mountains. Bot. Pac. J. Plant Sci. Conserv..

[5] Ismailova D.M., Nazimova D.I., Balzter H. (2010). Long-term dynamics of mixed fir-aspen forests in West Sayan (Altai-Sayan Ecoregion). Environmental Change in Siberia: Earth Observation, Field Studies and Modelling.

[6] Krestov P.V., Nazimova D., Stepanov N., DellaSala D., DellaSala D. (2010). Humidity-dependent forests of the Russian Far East, Inland Southern Siberia, and Korean Peninsula. Temperate and Boreal Rain Forest of the World: Ecology and Conservation.

[7] Lashchinsky N.N., Korolyuk A.Y. (2015). Syntaxonomy of zonal dark-coniferous forests of southern taiga of the West Siberian plain and of humid low-mountains of the Altai-Sayan mountain region. Veg. Russ..

[8] Elesova N., Silanteva M., Sokolova L. (2019). Rare plants and plant communities of the projected “Togul” National Park (Altai Region) BIO Web of Conferences. Results Prospect. Geobot. Res. Sib..

[9] Lugtenberg B., Kamilova F. (2009). Plant-growth-promoting rhizobacteria. Annu. Rev. Microbiol..

[10] Marilley L., Aragno M. (1999). Phylogenetic diversity of bacterial communities differing in degree of proximity of *Lolium perenne* and *Trifolium repens* roots. Appl. Soil. Ecol..

[11] Morgan J.A., Bending G.D., White P.J. (2005). Biological costs and benefits to plant-microbe interactions in the rhizosphere. J. Exp. Bot..

[12] Xiong Q., Hu J., Wei H., Zhang H., Zhu J. (2021). Relationship between plant roots, rhizosphere microorganisms, and nitrogen and its special focus on rice. Agriculture.

[13] Pascale A., Proietti S., Pantelides I.S., Stringlis I.A. (2020). Modulation of the root microbiome by plant molecules: The basis for targeted disease suppression and plant growth promotion. Front. Plant. Sci..

[14] Guerrieri A., Dong L., Harro J. (2019). Bouwmeester. Role and exploitation of underground chemical signaling in plants. Pest. Manag. Sci..

[15] Chaparro J.M., Badri D.V., Vivanco J.M. (2014). Rhizosphere microbiome assemblage is affected by plant development. ISME.

[16] Lundberg D.S., Lebeis S.L., Paredes S.H., Yourstone S., Gehring J., Malfatti S., Tremblay J., Engelbrektson A., Kunin V., Glavina T. (2012). Defining the core *Arabidopsis thaliana* root microbiome. Nature.

[17] Mendes L.W., Kuramae E.E., Navarrete A.A., van Veen J.A., Tsai S.M. (2014). Taxonomical and functional microbial community selection in soybean rhizosphere. ISME J..

[18] Beckers B., Op De Beeck M., Weyens N., Boerjan W., Vangronsveld J. (2017). Structural variability and niche differentiation in the rhizosphere and endosphere bacterial microbiome of field-grown poplar trees. Microbiome.

[19] Smalla K., Wieland G., Buchner A., Zock A., Parzy J., Kaiser S., Roskot N., Heuer H., Berg G. (2001). Bulk and rhizosphere soil bacterial communities studied by denaturing gradient gel electrophoresis: Plant-dependent enrichment and seasonal shifts revealed. Appl. Environ. Microbiol..

[20] Wei Z., Yang T., Friman V.-P., Xu Y., Shen Q., Jousset A. (2015). Trophic network architecture of root-associated bacterial communities determines pathogen invasion and plant health. Nat. Commun..

[21] Saleem M., Hu J., Jousset A. (2019). More than the sum of its parts: Microbiome biodiversity as a driver of plant growth and soil health. Ann. Rev. Ecol. Evol. System..

[22] Fierro-Coronado R.A., Quiroz-Figueroa F.R., García-Pérez L.M., Ramírez-Chávez E., Molina-Torres J., Maldonado-Mendoza I.E. (2014). IAA-producing rhizobacteria from chickpea (*Cicer arietinum* L.) induce changes in root architecture and increase root biomass. Can. J. Microbiol..

[23] Fahad S., Hussain S., Bano A., Saud S., Hassan S., Shan D., Khan F.A., Khan F., Chen Y., Wu C. (2014). Potential role 486 of phytohormones and plant growth-promoting rhizobacteria in abiotic stresses: Consequences for changing environment. Environ. Sci. Pollut. Res..

[24] Verma V., Ravindran P., Kumar P.P. (2016). Plant hormone-mediated regulation of stress responses. BMC Plant Biol..

[25] Bottini R., Cassan F., Piccoli P. (2004). Gibberellin production by bacteria and its involvement in plant growth promotion and yield increase. Appl. Microbiol. Biotechnol..

[26] Gutiérrez-Mañero F.J., Ramos-Solano B., Probanza A., Mehouachi J., Tadeo F.R., Talon M. (2001). The plant-growth-promoting rhizobacteria *Bacillus pumilus* and *Bacillus licheniformis* produce high amounts of physiologically active gibberellins. Physiol. Plant..

[27] USS Working Group WRB (2015). World Reference Base for Soil Resources 2014, Update 2015 International Soil Classification System for Naming Soils and Creating Legends for Soil Maps.

[28] Abakumov E.V., Loyko S.V., Istigechev G.I., Kulemzina A.I., Lashchinskiy N.N., Andronov E.E., Lapidus A.L. (2020). Soils of Chernevaya taiga of Western Siberia—Morphology, agrochemical features, microbiota. Agric. Biol..

[29] Polyakov V., Abakumov E., Lodygin E., Vasilevich R., Lapidus A. (2022). Distribution of molecular weight of humic substances isolated from soils of tallgrass temperate rainforests (Chernevaya taiga). Agronomy.

[30] Bazilevich N.I., Titlyanova A.A. (2008). Biotic Turnover on Five Continents: Element Exchange Processes in Terrestrial Natural Ecosystems.

[31] Tzollas N.M., Zachariadis G.A., Anthemidis A.N., Strati J.A. (2010). A new approach to indophenol blue method for determination of ammonium in geothermal waters with high mineral content. Int. J. Environ. Anal. Chem..

[32] Bates S.T., Berg-Lyons D., Caporaso J.G., Walters W.A., Knight R., Fierer N. (2011). Examining the global distribution of dominant archaeal populations in soil. ISME J..

[33] Martin V. (2011). Cutadapt removes adapter sequences from high-throughput sequencing reads. EMBnet J..

[34] Callahan B.J., McMurdie P.J., Rosen M.J., Han A.W., Johnson A.J.A., Holmes S.P. (2016). DADA2: High-resolution sample inference from Illumina amplicon data. Nat. Methods.

[35] Bolyen E., Rideout J.R., Dillon M.R., Bokulich N.A., Abnet C.C., Al-Ghalith G.A., Alexander H., Alm E.J., Arumugam M., Asnicar F. (2019). Reproducible, interactive, scalable and extensible microbiome data science using QIIME 2. Nat. Biotechnol..

[36] Quast C., Pruesse E., Yilmaz P., Gerken J., Schweer T., Yarza P., Peplies J., Glöckner F.O. (2012). The SILVA ribosomal RNA gene database project: Improved data processing and web-based tools. Nucleic Acids Res..

[37] Price M.N., Dehal P.S., Arkin A.P. (2010). FastTree 2—Approximately Maximum-Likelihood Trees for Large Alignments. PLoS ONE.

[38] Dhariwal A., Chong J., Habib S., King I., Agellon L.B., Xia J. (2017). MicrobiomeAnalyst—A web-based tool for comprehensive statistical, visual and meta-analysis of microbiome data. Nucleic Acids Res..

[39] Segata N., Izard J., Waldron L., Gevers D., Miropolsky L., Garrett W.S., Huttenhower C. (2011). 2011. Metagenomic biomarker discovery and explanation. Genome Biol..

[40] Frank A.K., Guzmán J.P.S., Shay J.E. (2017). Transmission of bacterial endophytes. Microorganisms.

[41] Wintz H., Fox T., Vulpe C. (2002). Responses of plants to iron, zinc and copper deficiencies. Biochem. Soc. Trans..

[42] Hauer-Jákli M., Tränkner M. (2019). Leaf magnesium thresholds and the impact of magnesium on plant growth and photo-oxidative defense: A systematic review and meta-analysis from 70 years of research. Front. Plant Sci..

[43] Vieira S., Sikorski J., Dietz S., Herz K., Schrumpf M., Bruelheide H., Scheel D., Friedrich M.W., Overmann J. (2020). Drivers of the composition of active rhizosphere bacterial communities in temperate grasslands. ISME J..

[44] Thiergart T., Durán P., Ellis T., Vannier N., Garrido-Oter R., Kemen E., Roux F., Alonso-Blanco C., Ågren J., Schulze-Lefert P. (2020). Root microbiota assembly and adaptive differentiation among European *Arabidopsis* populations. Nat. Ecol. Evol..

[45] Bastian F., Bouziri L., Nicolardot B., Ranjard L. (2009). Impact of wheat straw decomposition on successional patterns of soil microbial community structure. Soil Biol. Biochem..

[46] Boukhatem Z.F., Merabet C., Tsaki H. (2022). Plant growth promoting Actinobacteria, the most promising candidates as bioinoculants?. Front. Agron..

[47] Bünger W., Jiang X., Müller J., Hurek T., Reinhold-Hurek B. (2020). Novel cultivated endophytic Verrucomicrobia reveal deep-rooting 539 traits of bacteria to associate with plants. Sci. Rep..

[48] Lynch M.D.J., Neufeld J.D. (2015). Ecology and exploration of the rare biosphere. Nat. Rev. Microbiol..

[49] Jousset A., Bienhold C., Chatzinotas A., Gallien L., Gobet A., Kurm V., Kirsten Küsel K., Rillig M.C., Rivett D.W., Salles J.F. (2017). Where less may be more: How the rare biosphere pulls ecosystem strings. ISME J..

[50] Shade A., Hogan C.S., Klimowicz A.K., Linkse M., McManus P.S., Handelsman J. (2012). Culturing captures members of the soil rare biosphere. Environ. Microbiol..

[51] Davis K.E.R., Sangwan P., Janssen P.H. (2011). Acidobacteria, Rubrobacteridae and Chloroflexi are abundant among very slow-growing and mini-colony-forming soil bacteria. Environ. Microbiol..

[52] Sieber C.M.K., Paul B.G., Castelle C.J., Hu P., Tringe S.G., Valentine D.L., Andersen G.L., Banfield J.F. (2019). Unusual metabolism and hypervariation in the genome of *a Gracilibacterium* (BD1-5) from an oil-degrading community. MBio.

[53] Hanada S., Sekiguchi Y., Rosenberg E., DeLong E.F., Lory S., Stackebrandt E., Thompson F. (2014). The Phylum Gemmatimonadetes. The Prokaryotes.

[54] Daims H., Lebedeva E.V., Pjevac P., Han P., Herbold C., Albertsen M., Jehmlich N., Palatinszky M., Vierheilig J., Bulaev A. (2015). Complete nitrification by *Nitrospira* bacteria. Nature.

[55] Zhou Y., Lambrides C.J., Li J., Xu Q., Toh R., Tian S., Yang P., Yang H., Ryder M., Denton M.D. (2020). Nitrifying Microbes in the rhizosphere of perennial grasses are modified by biological nitrification inhibition. Microorganisms.

[56] Brodt S., Six J., Feenstra G., Ingels C., Campbell D. (2011). Sustainable Agriculture. Nat. Educ. Knowl..

[57] Glick B.R., Gamalero E. (2021). Recent developments in the study of plant microbiomes. Microorganisms.

[58] Kopittke P.M., Menzies N.W., Wang P., McKenna B.A., Lombi E. (2019). Soil and the intensification of agriculture for global food security. Environ. Int..

[59] Bulgarelli D., Schlaeppi K., Spaepen S., Ver Loren van Themaat E., Schulze-Lefert P. (2013). Structure and functions of the bacterial microbiota of plants. Ann. Rev. Plant. Biol..

[60] Basu A., Prasad P., Das S.N., Kalam S., Sayyed R.Z., Reddy M.S., El Enshasy H. (2021). Plant Growth Promoting Rhizobacteria (PGPR) as green bioinoculants: Recent developments, constraints, and prospects. Sustainability.

[61] Nielsen U.N., Wall D.H., Six J. (2015). Soil biodiversity and the environment. Ann. Rev. Environ. Res..

[62] Graham E.B., Knelman J.E., Schindlbacher A., Siciliano S., Breulmann M., Yannarell A., Nemergut D.R. (2016). Microbes as engines of ecosystem function: When does community structure enhance predictions of ecosystem processes?. Front. Microbiol..

[63] Wagg C., Schlaeppi K., Banerjee S., Kuramae E.E., van der Heijden M.G.A. (2019). Fungal-bacterial diversity and microbiome complexity predict ecosystem functioning. Nat. Commun..

